# Optimization of Biological Synthesis of Silver Nanoparticles using *Fusarium oxysporum*

**Published:** 2013

**Authors:** Hassan Korbekandi, Zeynab Ashari, Siavash Iravani, Sajjad Abbasi

**Affiliations:** a ^a^*Geneticsand Molecular Biology Department, School of Medicine, Isfahan University of Medical Sciences, Isfahan, 81744-176, Iran.*; b *Biotechnology Department, Faculty of Pharmacy and Pharmaceutical Sciences, Isfahan University of Medical Sciences, Isfahan, Iran.*; c *Faculty of Pharmacy and Pharmaceutical Sciences, Isfahan University of Medical Sciences, Isfahan, Iran. *

**Keywords:** Silver nanoparticles, *Fusarium oxysporum*, Optimization, Biosynthesis, Intracellular synthesis GABA_A_ receptor

## Abstract

Silver nanoparticles are increasingly used in various fields of biotechnology and applications in the medicine. Objectives of this study were optimization of production of silver nanoparticles using biotransformations by *Fusarium oxysporum*, and a further study on the location of nanoparticles synthesis in this microorganism. The reaction mixture contained the following ingredients (final concentrations): AgNO_3_ (1-10 mM) as the biotransformation substrate, biomass as the biocatalyst, glucose (560 mM) as the electron donor, and phosphate buffer (pH= 7, 100 mM). The samples were taken from the reaction mixtures at different times, and the absorbance (430 nm) of the colloidal suspensions of silver nanoparticles hydrosols was read freshly (without freezing) and immediately after dilution (1:40). SEM and TEM analyses were performed on selected samples. The presence of AgNO_3_ (0.1 mM) in the culture as enzyme inducer, and glucose (560 mM) as electron donor had positive effects on nanoparticle production. In SEM micrographs, silver nanoparticles were almost spherical, single (25-50 nm) or in aggregates (100 nm), attached to the surface of biomass. The reaction mixture was successfully optimized to increase the yield of silver nanoparticles production. More details of the location of nanoparticles production by this fungus were revealed, which support the hypothesis that silver nanoparticles are synthesized intracellularly and not extracellularly.

## Introduction

Developments in the organization of nanoscale structures into predefined superstructures ensure that nanotechnology will play critical role in many key technologies (1-2). Nowadays, nanotechnology and nanoparticle synthesis are among the interesting scientific areas, and there is growing attention to produce nanoparticles using eco-friendly methods (green chemistry) (3- 6). Nanoparticles have been produced physically and chemically for a long time,however, recent developments show the critical role of microorganisms and biological systems in the production of metal nanoparticles. The use of organisms in this area is rapidly developing due to their growing success and ease of formation of nanoparticles. Moreover, biosynthesis of metal nanoparticles is an environmentally-friendly method (green chemistry) without use of harsh, toxic and expensive chemicals.For instance, production of silver nanoparticles by chemical reduction (*e.g.*, hydrazine hydrate, sodium borohydride, DMF, and ethylene glycol) may lead to absorption of hazardouschemicals on the surfaces of nanoparticles raising the toxicity issues. Despite stability and a green method of formation, the nanoparticle synthesis rate is not comparable to non-biological synthesis methods. In reality, biosynthesis would have greater commercial acceptance if the nanoparticles could be synthesized more rapidly and economically on a large scale. However, in order to achieve better control of size, morphology, stability, and rate of nanoparticle preparation, biological methods could be used with some optimization. Various physical and chemical methods have been used for the synthesis of metal nanoparticles, however,the ability of organisms in production of metal nanoparticles with desired morphological characteristics and sizes has opened a new exciting approach toward the nanoparticle synthesis (5, 7-10). The production of nanoparticles using organisms and the most promising systems and conditions in nanoparticle biosynthesis has been previously reported (11, 12). 

The objectives of recent studies were better control of morphology and size of nanoparticles, as well as the rate of production. In order to provide a controlled and up-scalable process for the synthesis of highly stable nanoparticles, reaction conditions such as pH, light, temperature, buffer strength, electron donor and its concentration, biomass and substrate concentration, mixing speed, and exposure time should be optimized (9, 11-16). One of the most promising organisms used in nanoparticle production is *Fusarium oxysporum *(8). Theuse of *F. oxysporum *as a source of enzymesthat can catalyze specific reactions leading to inorganicnanoparticles is a rational biosynthesis strategy. This organism has shown great potential in bioreduction and bioremediation of metal ions. It has been demonstrated that stable silver nanoparticles (5- 50 nm) could be producedwhen *F. oxysporum*is exposed to aqueous Ag^+^ ions (19). Synthesis of nanoparticles using *F. oxysporum*has many advantages suchas ease with which the process can be scaled up, economicviability, possibility of easily covering large surfaceareas by suitable growth of the mycelia, *etc*. Theshift from bacteria to fungi has the added advantage thatdownstream processing and handling of the biomasswould be much simpler. Compared to the bacterial fermentations, in which the process technology involves the use ofsophisticated equipment for getting clear filtrates fromthe colloidal broths, fungal broths can be easily filteredby filter press of similar or simple equipment, thus savingconsiderable investment costs.Moreover, compared to bacteria, *F. oxysporum*is known to secrete much higher amountsof proteins, thereby significantly increasing the productivityof the biosynthetic approach (19-23).Several studies have been conducted for theproduction of nanoparticles using *F. oxysporum*, but these qualitative studies have not been optimized (8, 19-23). It has been claimed that this production is extracellular. The objectives of this study were optimization of silver nanoparticles production and studying the exact location of their synthesis. 

## Experimental

*F. *oxys*porum *(DSM 841) was purchased from DSMZ (Germany) as active culture and revived on MGYP broth. Standard calibration curve of OD (550 nm) of a well grown culture was plotted against the biomass dry weight to access an easy and quick assay for the determination of biomass by OD reading. Growth curve of the microorganism in presence of AgNO_3_ (0.1 mM), as the enzyme inducer, was plotted to estimate the optimum harvesting time. The culture was centrifuged (3080 *g*, 15 min, Gallenkamp centrifuge 200) to harvest the biomass at the late exponential phase. The reaction mixture contained the following ingredients (final concentrations): AgNO_3_ (1-10 mM) as the biotransformation substrate, biomass (0.157, 0.557, 2.33 and 4.96 g _dry− weight_ L^−^^1^) as the biocatalyst, glucose (56 mM) as the electron donor, and phosphate buffer (pH=7, 100 mM). Effects of different factors, including inducer, electron donor, substrate concentration, and biomass concentration were examined by using one factor at a time method. The aforementioned ingredients were added in appropriate volumes into Duran^®^ bottles (100 mL) and were incubated (80 rpm, 25°C). Samples (1.5 mL ×3) were taken from the reaction mixtures at different times, and the absorbance (430 nm) of the colloidal suspensions of silver nanoparticles (hydrosols) was read freshly (without freezing) and immediately after dilution (1:40). Absorption spectra were measured on a Shimadzu (UVmini-1240) spectrophotometer. Scanning electron microscope (SEM) and transmission electron microscopy (TEM) were performed on selected samples in order to investigate the process of formation of silver nanoparticles and study the size and shape of them. Furthermore, TEM was performed to study the exact location of the silver nanoparticles synthesis in relation to the *F. oxysporum*cells. SEM images of the sample were obtained using XL-30 Phillips scanning electron microscope. Samples for TEM were prepared by drop-coating the Ag nanoparticle solutions onto carbon-coated copper grids. Micrographs were obtained using EM 201 Phillips transmission electron microscope.

## Results and Discussion

*Visual inspection*

When *F. oxysporum *biomass was exposed to Ag^+ ^ions (AgNO_3_, 1 mM), the color of the reaction mixture turned to yellowish brown and then dark brown, which was in agreement with the previous studies and was considered as the production of colloidal suspension (hydrosol) of silver nanoparticles (24, 25). The appearance of dark brown is due to the excitation of surface plasmon resonance in the nanoparticles.

*Growth curve of F. oxysporum*

**Figure 1:**
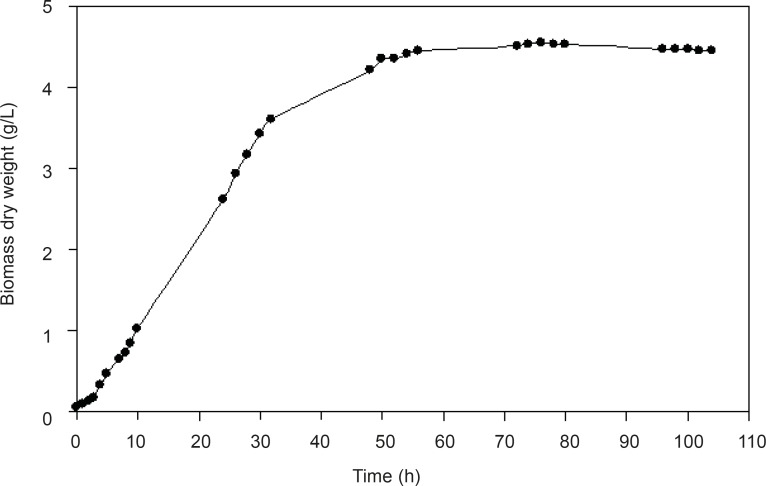
Growth curve of *F. oxysporum *on MGYP broth- Optical density of the culture was measured during the fermentation time and converted to dry weight

In order to determine the optimum time to harvest the cells, the growth curve of *F. oxysporum*in presence of the enzyme inducer, AgNO_3_ (0.1 mM), was studied (Figure 1). In most enzymatic reactions, induction of the responsible enzymes increases activity. Therefore, we decided to induce the enzymes that seemed to be responsible for the reduction of the substrates. Silver nitrate in a low concentration (0.1 mM) was chosen as the inducer. There was no lag phase in growth curve, and in fact the acceleration phase of the reaction was between 0-4 h. Logarithmic or exponential growth phase was between 4-32 h. Between hours 32-55 and 55-105, deceleration and stationary phases were observed, respectively. The optimum harvest time could be between hours 28 and 32.

*Monitoring synthesis of silver nanoparticles*

**Figure 2:**
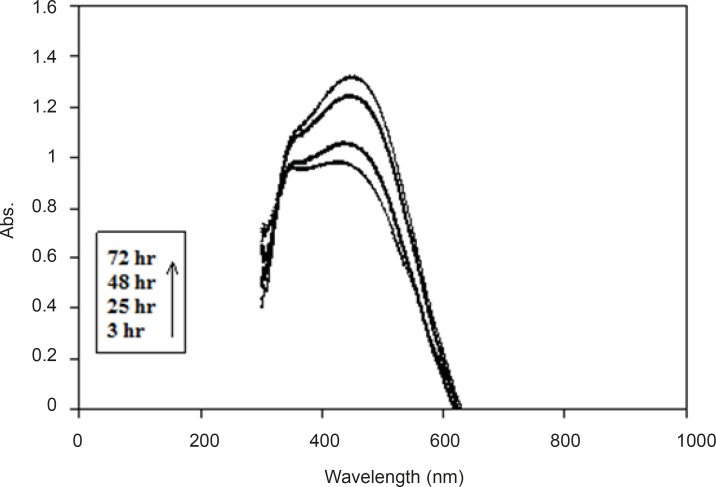
UV/Vis absorption spectrum of the produced colloidal Ag.The spectrum was obtained at different time points after the start of AgNO_3_ (1 mM) reduction using *F. oxysporum *previously induced with AgNO_3_ (0.1 mM).

We examined the UV/Visabsorption spectrum of colloidal silver to monitor the biosynthesis of silver nanoparticles. Ultraviolet visible absorption spectra of samples were evaluated at different times after the start of the reaction. The λ_max _was about 430 nm in all samples. During the reaction period (from 3 h to 72 h), an increase in absorbance was observed in this wavelength, which is likely due to the increase in production of colloidal silver nanoparticles (Figure 2). Thus, the absorption spectrums of silver hydrosols obtained in previous studies were confirmed (8, 19). 

*Observations regarding preparation of samples for analysis*

In the samples, separation of biomass eliminated the interference in reading UV/Vis absorbance (430 nm) of colloidal silver nanoparticles. It was assumed that sample filtration with filter discs (0.2 μm) or by centrifugation (15294 *g*, Eppendorf centrifuge 5417 R) could be useful. However, after tryingboth of these methods, the absorbance (430 nm) of colloidal suspension of nanoparticles decreased greatly to near zero, and the color (yellow to brown) disappeared. It might be due to the aggregation of nanoparticles or their entrapment in the biomass. Therefore, filtering and centrifuging the samples showed negative effects on nanoparticles and should be avoided. Instead of these two methods, samples were diluted (dilution factor = 40) and their absorbance was read at 430 nm, and corrected for the absorbance before the start of the reaction.

*Analysis of enzymatic reaction of nanoparticle synthesis*

**Figure 3:**
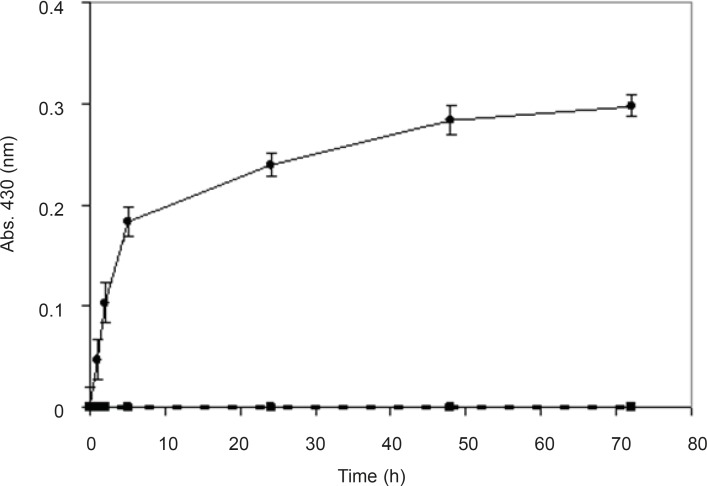
Time course of biotransformation of AgNO_3_ using non-boiled (●) and boiled (○) biomass. The spectrum was obtained at different time points after the start of AgNO_3_ (1 mM) reduction using *F. oxysporum *previously induced with AgNO_3 _(0.1 mM) (*n*-value= 3).

In order to prove that the synthesis of nanoparticles is an enzymatic reaction and is not chemical, biological mass was boiled for 15 min until the fungi were killed and their enzymes were denatured. Nanosilver particles were produced from the reaction mixture containing the biomass, but no absorbance (430 nm) was observed from the reaction mixture with boiled biomass (Figure 3). It seems that boiling the biomass inhibited the nanoparticles production, which supports the hypothesis that the nanoparticle biosynthesis is enzymatic, and the enzymes secreted from the microbial cells are responsible for the reduction of silver ions to silver nanoparticles. Further, these results are in agreement with previous studies (8, 19-23).

*Factors affecting the biosynthesis of metal nanoparticles*

The main challenges frequently encountered in the biosynthesis of nanoparticles are thecontrol ofshape and size of the nanoparticles as well as to achieve the mono-dispersity in solution phase. There are several factors which may directly influence or cause some hindrance in synthesis of metal nanoparticles. Therefore, we examined effects of different factors, including inducer, electron donor, substrate concentration, and biomass concentration. 

*Effect of inducer*

**Figure 4:**
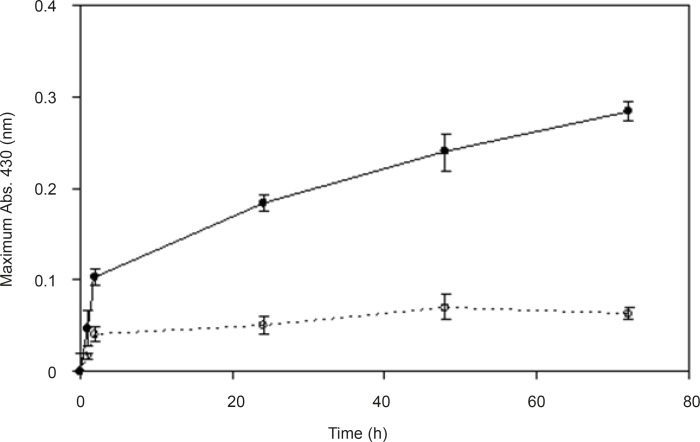
Effect of previous exposure of the culture to inducer-The spectrum was obtained at different time points after the start of AgNO_3_ (1 mM) reduction using *F. oxysporum *cells which were previously induced (●), or not induced (○) with AgNO_3_ (0.1 mM) (*n*-value = 3).

In order to understand the effect of inducer on enzyme activity and biosynthesis of nanoparticles, silver nitrate (0.1 mM) was used as the enzyme inducer. Two series of samples were collected from the reaction mixtures (with and without the inducer). In the reaction mixture without silver nitrate, a dramatic decline in the absorbance was observed (Figure 4). We hypothesize that pretreatment with the silver nitrate (as the inducer) triggers the transcription of genes involved in the synthesis of reducing enzymes (gene expression) and can increase the productivity of nanoparticle synthesis. Thus, the presence of silver nitrate as an enzyme activity inducer seems to be important for scaling up this process.

*Effect of electron donor*

**Figure 5:**
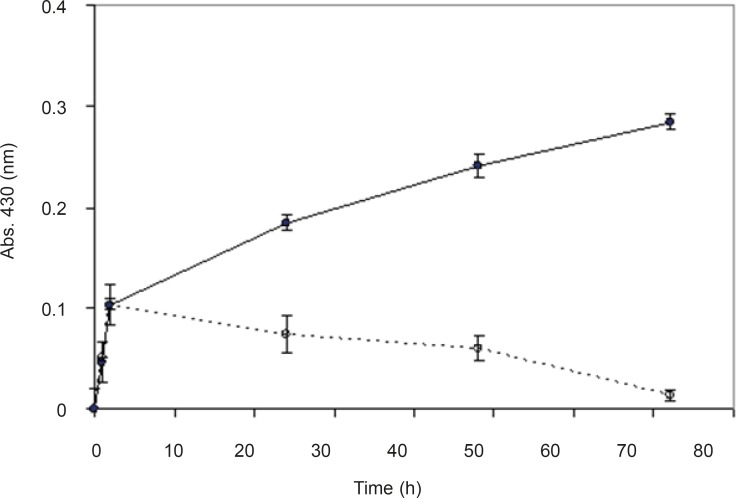
Effect of electron donor on nanoparticle production**- **The spectrum was obtained at different time points after the start of AgNO_3_ (1 mM) bioreduction using *F. oxysporum *silver nitrate (0.1 mM, as the inducer) in the presence (●) and absence (○) of electron donor (glucose) (*n*-value = 3).

For investigating the effect of electron donor in the process of nanoparticle synthesis and bioreduction of silver nitrate (AgNO_3_), glucose (as the electron donor) was added to the reaction mixture. Hypothetically, electron donors provide electrons for reduction of cofactors, and make the reduction of silver ions (Ag^+^) to silver metal (Ag^0^) faster. Therefore, two series of samples were collected from the reaction mixtures (with and without glucose), and time course of the biotransformation of AgNO_3_ using whole cells of *F. oxysporum *was plotted. In both presence and absence of glucose (56 mM) the reduction started quickly (Figure 5). In the presence of glucose, the reaction rate decreased after 2 h however,absorbance continued to increase gradually until 48^th^hour. However, in the reaction mixture without glucose, after about 2 h, the absorbance gradually declined to near zero at 48^th^h. Glucose (56 mM) as the electron donor had positive effects on nanoparticle production. It seems that in the absence of the electron donor, electron reservoirs in reaction mixture for the recovery and revival of cofactors, after a short time (two h) are finished and the reaction declines. Therefore, we decided to use glucose as the electron donor in all processes of nanoparticle production by using *F. oxysporum*. 

*Effect of substrate concentration*

**Figure 6:**
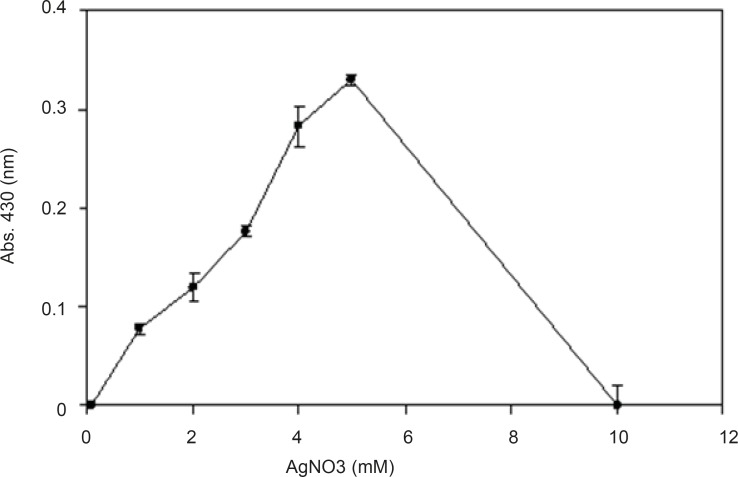
Effect of different concentrations of the substrate**- **Absorption spectra of maximum production of silver nanoparticles against various concentrations of AgNO_3_ (0.1, 1, 2, 3, 4, 5, and 10 mM) was read and recorded (*n*-value = 3).

In biotransformations, one of the factors making the reaction more economical and efficient is finding the maximum concentration of substrate which could be converted to the final product. Therefore, we investigated different concentrations of silver nitrate in the reaction mixture in order to evaluate the maximum concentration of the substrate for nanoparticle production (Figure 6). By gradual increase in concentration of AgNO_3_ to 5 mM, the nanoparticle production was increased, however,by further increasing to 10 mM, the production decreased. It might be interpreted that this amount of AgNO_3_ has toxic effects on *F. oxysporum *biomass (the biocatalyst), and the optimum concentration of silver nitrate should be around 5 mM.

*Effect of biomass concentration*

It appears that the organism’s enzymes (the biocatalysts) are the major agents in biological synthesis of nanoparticles. The biocatalysts can be used as whole cells, crude enzymes, and purified enzymes. In this study we used whole cells. Although the rates of bioreduction by using cell extract or purified enzyme are faster, these two methods need coenzymes (such as NADH, NADPH, FAD, *etc*.) for the continuation of the reaction. As they are expensive, the use of whole cells is preferred because the coenzymes will be recycled during the pathways in intact live cells. 

The mechanism which is widely accepted for biosynthesis of silver nanoparticles is the presence of enzyme “Nitrate reductase”.Nitrate reductase is an enzyme in the nitrogen cycle responsible for the conversion of nitrate to nitrite (26-28). The reduction mediated by the presence of the enzyme in organisms has been found to be responsible for the synthesis. It was investigated that the exposure of Au and Ag ions to *F. oxysporum *resulted in release of reductase (nitrate reductase) and subsequent formation of highly stable gold and silver nanoparticles in solution (8). Nitrate reductase was essential for ferric iron reduction and it seems that fungi which have this enzyme are able to reduce gold and silver ions too. 

**Figure 7:**
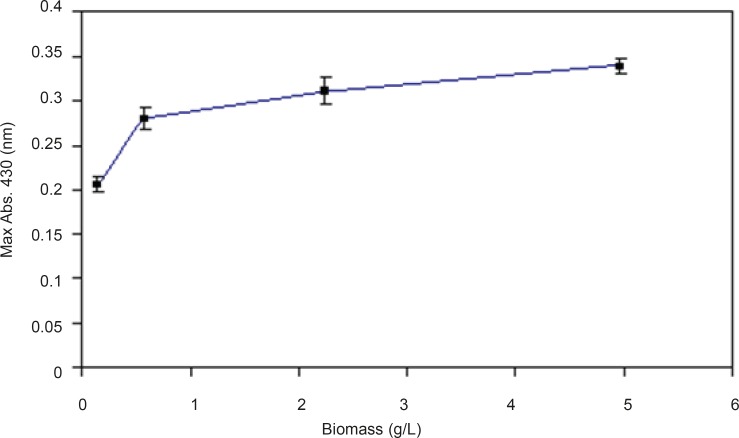
Effect of different biomass concentrations- Effect of different maximum absorption of silver nanoparticles against various concentrations of biomass (0.157, 0.558, 2.23, and 4.96 gL^-1^) was read and recorded (*n*-value = 3).

In optimization of reaction conditions one of the efforts would be finding the appropriate concentration of biomass which can produce maximum products. Different concentrations of the biomass were prepared and the effect of this parameter was evaluated.By increasing the biomass concentrations, nanoparticles production was also increased but this relationship was not linear (Figure 7). 

*Analysis of silver nanoparticles with SEM and TEM*

**Figure 8:**
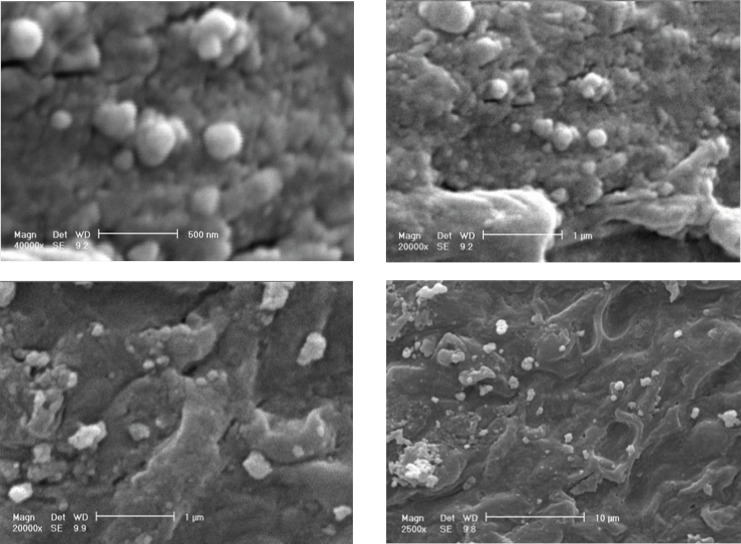
SEM micrographs of silver nanoparticles produced by the reaction of 1 mM AgNO_3_ solution with *F. oxysporum*

**Figure 9:**
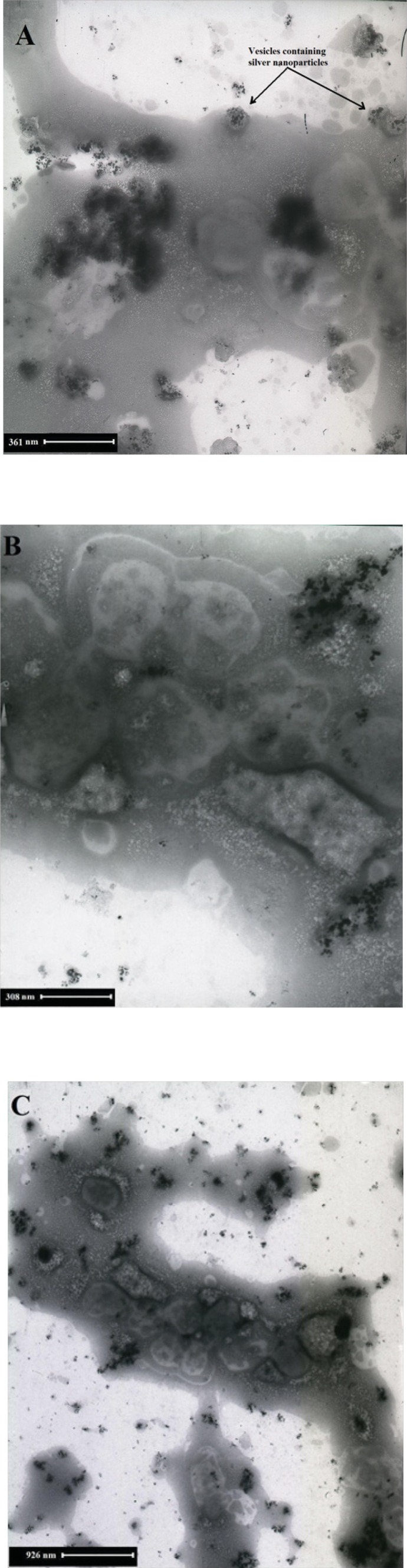
TEM micrographs recorded from a drop-coated film of an aqueous solution of Ag^+ ^ions incubated with *F. oxysporum*- Magnification are (A: 10 × 12000), (B: 10 × 20000) and (C: 10 × 7000).

Figure 8 shows SEM micrographs of Ag nanoparticles produced by the reaction of AgNO_3_ solution with *F. oxysporum *biomass after 48 h in the presence of inducer and electron donor. Silver nanoparticles were almost spherical, single (25- 50 nm) or in aggregates (100 nm), attached to the surface of fungal cells. By TEM micrographs from the same batch analyzed by SEM, it was shown that the nanoparticles are produced inside the cytoplasm, and are aggregated in vesicles, which are then secreted through cell membrane by exocytosis (Figure 9). 

## Conclusions

The present study demonstrated the bioreductive synthesis of silver nanoparticles using *F. oxysporum*. Previous researchers reported qualitative production of silver nanoparticles by *F. oxysporum, *but they did not optimize the reaction mixture. In this study, and due to our experience in optimization of biotransformation reactions, the reaction mixture was successfully optimized to increase the yield of nanoparticles production. The optimum conditions were as follows: biomass concentration (4.96 g _dry−weight_ L ^−1^), substrate concentration (AgNO_3_, 5 mM), presence of electron donor (glucose, 56 mM), presence of inducer (AgNO_3_, 0.1 mM). Thus, the necessary information for economical production of silver nanoparticles by *F. *oxys*porum *is now more complete. The reduction of metal ions and stabilization of the silver nanoparticles was confirmed to occur by an enzymatic process. It seems that the first step involves trapping of the Ag^+^ ions by *F. oxysporum *cells. More details of the location of nanoparticles production by this fungus were revealed, and the previous theories were corrected. In contrast with the previous studies, we suggest that the nanoparticle production in *F. oxysporum *is intracellular by engulfing the nanoparticles in vesicles, transporting, and excreting of them through exocytosis outside of the cells. 
